# Supplementary material for: Chromothripsis during telomere crisis is independent of NHEJ, and consistent with a replicative origin
Source: Genome Res. 2019 May;29(5):737–49. doi: 10.1101/gr.240705.118 (PMC6499312; doi:10.1101/gr.240705.118)
Supplement: Supplemental Material [file supp_gr.240705.118_Supplemental_file_1.zip › contigs/annotated_contigs/DB78/contig.4.DB78_length_599_mean_cov_15.0250417362.docx]

**DB78_length_599_mean_cov_15.0250417362**

```
ATTCCATTCGTGTCCATTACATTCCTTTCCATTCCATTCGCGTCTGTTCCATTCCATTCGATTCCAGTCCATT|TAAT|TCAGCTGGGG
 >chr1:224203007-224203080 - E=4e-05                                          >chr22:48
ACTAAGCTGGAGAGACAGGAGG|CTG|ACAGTCTCACACACAGTAGGTGCTGGATAGTTCTAATGAGATTCTTAGACAAATGTGAAGTG
723928-48723963 - E=2e-09 >chr22:48708511-48708696 + E=1e-96
AAAATCTGTAAGCTCCCTATGAGTAGGGTGTCTGCAAAGTTCCCTAGTTCATCAGCAGTGTGTGGATTGCAGGATCTGCAGCATTAAAT

GGGTTTAGGAACTATTCCATGGACAGGTGAG|TAT|AATACTATTAAAACATAACAAGTGGGTAGTAGTGACTGCAGTAAAATTGTCTA
                                   >chr22:48684780-48685082 + E=5e-167
AAAATCACTTCATGGGTCAATAATAAGAAAAGATTGAGACACATCAGTATATGGACAGGCTTTCCCACAGGTGACAACAAGATTCTATC

CTTCTAGGAGGTCAAGTTCAGGTAGAAGGCAACACCCCTTCCTAATGGTCCAGCCAAAAGTCCTAGGGCTGCCTCTCAGCCAGCAGGTT

TGATCCTGGGCCTAGCACTGTGTCTCTGAGTAACCAGGATTTAGGCATATGATCATGCTGGGGCTTTGGGG
```
